# Mr. Evans's Pathological and Practical Remarks on Ulcerations of the Genital Organs

**Published:** 1820-06-01

**Authors:** 


*Pathological and Practical Remarks on Ulcerations of the Genital Organs, pointing out the Characters by which they may be discriminated, shewing the consecutive Diseases to which they give rise; and containing an Inquiry into the Use of Mercury in their Treatment.* By JAMES EVANS, Surgeon of His Majesty's 57th Regiment. One vol. 8vo. pp. 128. London, 1819.

IT is said, that "Nature is always the same." We very much doubt the truth of this assertion. There is nothing fixed or stationary in any part of the solar system, as far as we can judge by distant examination; and we are quite sure that the earth, and all which it inhabit, are perpetually undergoing changes and revolutions. This mutability is not more prominent in the material than in the intellectual world. Religion, morals, politics, are so continually changing, that it is hazardous to proclaim TRUTH itself as immutable; at least, what passes for truth in this world. It is not to be supposed that PHYSIC offers an exception to so general a law—far from it! Not only does the science itself revolve as a whole, but all its parts circle round their centres, like the earth in its gyrations round the sun. The Sophists of Athens did not fail to note this fluxionary state of things, and the Disciples of Protagoras asserted (with more truth than on many other occasions) that no individual ever saw the *same* thing twice; and consequently, that no two individuals, in succession, could possibly see an identical object. This sophism, if it be one, appears now to be verified in medicine. Where can we find two physicians agree about what is, or is not, the small-pox?—where two surgeons who can unanimously decide on what is, or is not, the great pox? Remedies must, of course, follow the fate of diseases. Accordingly, we have the vaccinists and anti-vaccinists—mercurialists and anti-mercurialists. Whether these schisms in medicine may terminate in scepticism, as they have in religion, we know not; but, in both cases, much confusion is likely to prevail, before we have—*lucem ex fumo*.

The establishment of a correct diagnosis between syphilitic and syphiloid diseases is unquestionably a leading step towards reconciling the jarring opinions and practices which have hitherto prevailed in this department of surgery; consequently, every man who endeavours to effect this des rable object, has a claim on our gratitude, and need not anticipate censure from any critic, whose censure or applause could have any influence. We shall therefore proceed in our analytical labours, without further preface.

Mr. Evans divides his work into two Books, one on those diseases which have, the other on those affections which have *not* arisen from sexual intercourse; to which is added, an Appendix that shall be noticed in due time. The Books are subdivided into Chapters, corresponding to the number of diseases each Book contains. Mr. Evans also classes his observations under the heads "Description, Diagnosis, Causes, and Treatment;" which is, perhaps, as lucid and useful an arrangement as he could have chosen.

### Book I. Diseases arising generally, or entirely independent of sexual intercourse.

### *Chap.* 1. *Phlegmon.*

*Description.* Most commonly situated in the loose skin covering the glands; its progress exactly resembling that of phlegmon in any other part of the body. After it suppurates and bursts, Nature endeavours to get rid of the loose integuments forming the sides of the sac by *ulceration*, which has such a spreading appearance, and unhealthy surface, as to cause much alarm. The sore, however, does not increase in depth.

*Diagnosis.* The progress to suppuration, the flow of matter on ulceration taking place, and the immediate diminution of size, after the discharge, well distinguish phlegmon of the penis from other affections.

*Treatment.* In phlegmon on the *prepuce*, cold applications are ineffectual; but they are useful when the body of the penis is the seat of the disease. In the *former* case, then, fomentations, warm emollient poultices, an early exit to the matter when formed, and a removal of the whole covering of the abscess by the knife, or potassa fusa, should the ulcerative process be set up, constitute the best methodus medendi.*

* A cotemporary critic seems to question the propriety of our author's introducing phlegmon of the penis, since the disease is common to all other parts of the body's surface. We conceive, however, that if a man

*Chap.* 2. *Anthrax.*

It is difficult to distinguish this formidable affection of the membrum virile, at first, from phlegmon. But when a lancet is pushed into it, instead of a flow of matter, only a few drops of blood and serum escape, followed soon afterwards by a projection of diseased cellular membrane, of a yellow colour. Nature endeavours to free herself from this disease by ulceration, or the sloughing process. Two valuable cases of this disease are detailed by our author.

*Treatment.* Warm emollient poultices; attention to the state of the skin and bowels; venesection, if much febrile action be present, and early opening, to give vent to the contents of the tumour.

*Chap.* 3. *Tubercula.*

Mr. Evans has met with two kinds of these; one resembling what Dr. Bateman terms "Moluscum," which is a circular, and rather flattened tumour, of the same colour as the surrounding integuments, and slightly elevated. If a lancet be pushed into it, a cream-like fluid is found within. Unless they inflame, they are not troublesome, and sometimes continue for years without exciting much attention, being considered as warts. When inflammation supervenes, they sometimes shrink up and disappear, leaving the part divested of cuticle, or occasionally ulcerous, and troublesome to heal. The other affection of this kind, is a small inflamed tumour, sometimes flattened like the former, sometimes spherical. Instead of shrinking up, when inflamed, it forms a small pustule, succeeded by a scab, which is found covering a cup that has been the lower half of the sac.

*Treatment.* Mr. Evans would not like to interfere with the *first* form while in its indolent stage, unless urged by the patient, when he would endeavour to excite inflammation, in imitation of the manner in which nature removes the complaint. In the *second* form, after the subsidence of inflamundertakes to describe all the diseases of an organ, he has no right to exclude those which are common to other parts, as well as to the organ treated of. Moreover, in the case in question, phlegmon, we see, is modified by the peculiar structure of the penis and its coverings; consequently, it demands a modified treatment. It is our opinion, therefore, that Mr. Evans would *not* "have acted judiciously in discarding it from the list of diseases of the penis."†

† *Edinburgh Journal, January* 1820.

mation, Mr. E. has sometimes found it necessary to destroy the base by repeated applications of caustic, before the sores would heal.

### *Chap.* 4. *Herpes Preputialis.*

Excepting when it takes place on the *inner* surface of the prepuce, it seldom requires surgical assistance, unless from friction of the clothes or improper interference. In such case, the scab is partially or entirely removed, exposing an ulcer with a yellow, or white and plain surface; in shape frequently angular, and with abrupt edges.

"It is, however, when situated upon the inner surface of the prepuce, that the patient's fears are most strongly excited, and the practitioner too often deceived; the circumstances of its not scabbing when it occurs in that place, of the vesicles when broken, forming each a small circular ulcer with a white or yellow surface, and of their sometimes running quickly one into the other, with the tediousness of the healing process when interfered with, have, I much fear, in my own practice, very often occasioned this disease to be mistaken, and treated erroneously." 27.

*Diagnosis.* Herpes preputialis may be distinguished from venerola vulgaris (hereafter to be described) by its first appearing as a *cluster of vesicles,* whereas ven. vulg. appears as a *single pustule.* The scab in the *former* is little more than a scale; in the *latter,* it is generally of considerable size and thickness. When situated, however, on the *inner* surface of the prepuce, where no scab is formed, the diagnosis is not so easy. It has not the elevated edge and surface which venerola vulgaris exhibits about the eighth day.

*Cause.* Derangement of the digestive organs in general.*

The *Treatment* is very simple, as a weak solution of acetate of lead to allay inflammation.

### *Chap.* 5. *Psoriasis Preputialis.*

"This disease appears in the form of deep cracks or chasms around the margin of the prepuce, which (as is the case when the same disease affects the lip,) are extremely irritable, and apt to bleed whenever any attempt is made at retraction, but which, from the loose cellular texture of the prepuce are, in this case, generally much deeper: the discharge is of a glutinous nature until the mor-

* The Edinburgh Reviewer remarks, on Mr. Evans' Etiology of Herpes Preputialis, that *stricture* is a frequent cause of the disease.

bid action ceases, when it becomes purulent, and then the healing process begins, which is often very tedious." 31.

This disease is apt to occasion bubo; and Mr. E. has *not* met with it in persons whose foreskin did not naturally cover the glans.

The *Treatment* is diluted unguentum hydrargyri nitratum.

### *Chap.* 6. *Ulcus Erraticum.*

This ulcer begins with a small pustule, generally on the outer skin of the body of the penis, which bursts, and forms a scab; which, if allowed to remain, enlarges, with the ill-conditioned sore that is discovered on its removal. This ulcer, if of long standing, is marked by unhealthy granulations, alternating with foul excavations.

"When it occurs upon the *body of the penis*, it extends itself upwards, in a line partaking more or less of the spiral or circular form, sometimes healing below, while it continues to ulcerate above, its edge being generally somewhat raised and thickened, and oftentimes everted.

"When it occurs on the *pubis*, it sometimes forms distinct ulcerations, which spread equally, or nearly equally, on all sides: at other times, they will extend in circles, leaving in their centres insulated portions of integument, or, in some cases, healthy granulations will be thrown up from the bottom of the sore, perhaps near its centre, on which points the healing process shall commence.

"The pain attending this sore, which is frequently severe, the patient often describes as burning." 34.

*Cause.* In Mr. Evans's experience, a long-continued, irregular, or injudicious use of mercury; or a considerable previous derangement of the system.

*Diagnosis.* "In its early stages, [the pustular, and beginning of the ulcerative] this disease may be readily confounded with venerola vulgaris; but, after a time, the absence of the elevated surface, with the continuance of the ulcerative process, the alternate granulations and excavations, and the unequal extension of the sore (the ulceration perhaps spreading on one side, while it shall have stopped on the other, where healing may have commenced) will suffice to distinguish them; and for no other disease herein spoken of is this ulcer likely to be mistaken." 40.

*Treatment.* This is a very unmanageable sore: the same treatment that appears to agree to-day, will frequently disagree to-morrow. The following are the applications which Mr. Evans found most useful upon the whole. A lotion of from five to ten drops of nitric acid in a pint of pure water; sulph. zinci gr. ß to gr. iij, in an ounce of water; superacet. plumbi gr. ß to an ounce of water; the same proportion of argentum nitratum; ditto of the oxymur. hydrargyri; ungt. hyd. nit. ʒj; cerat. ceræ ʒiij. M. ft. unguentum. Such are the local means. If the patient be plethoric, he should be enjoined abstinence in diet, and to avoid wine, spirits, and malt liquor, and *vice versa.* The mineral acids and sarsaparilla are occasionally useful;

"But the remedy from which there has appeared the most decided good effects, was the blue pill, given in small quantities, so as to act gently upon the bowels, but not continued so long as to touch the mouth, especially when conjoined with occasional small doses of aperient salts."

This ends the first Book, or class of genital affections—*sine concubitu.*

### Book II. Diseases generally or entirely arising from sexual intercourse.

### *Chap.* 1. *Excoriatio.*

This, when recent, appears in irregular patches, with itching, increased and altered secretion, which, if the excoriation does not soon heal, becomes purulent, the abraded surfaces assuming a yellow colour. Although our author has not seen extensive ulceration arise, in the first instance, from mere excoriation; yet he can readily believe that such may be the case in the lower ranks of life, among persons of bad constitutions and uncleanly habits.

The prepuce and glans generally participate in this excoriation, and buboes are a common consequence.

*Cause.* The simplest is *direct* abrasion of the cuticle from mere *friction;* but such cause is so rare, that our author never met with one well-attested instance of it.

"The next case in point of infrequency, is where the disease takes place from too often repeated and long-continued friction, of which the denudation becomes an *indirect* consequence, it being preceded by patches of minute vesicles, which, breaking, leave the parts excoriated." 49.

A more frequent cause, our author thinks, is, "a greatly increased secretion of the sebaceous fluid, which, in these cases, has the appearance of cream; and which, unless prevented by frequent ablution, or certain applications, will soon give rise to the disease." Some people are peculiarly liable to this mal-secretion, and it commonly is dependent on derangement of the primæ viæ, resulting from intemperance. Neglect of cleanliness, however, would appear to lead to it in any constitution.

"But among all the causes of this disease, by far the most frequent is impure coition, by which an acrid secretion being applied, irritation, vesication, aud subsequent excoriation are produced.

"The altered secretion which in one person, will only produce those superficial ulcerations, which from their wanting certain peculiarities, are included under the head of Excoriation, will in another give rise to gonorrhœa, or venerola, or both. This circumstance, with there appearing to be a regular gradation up to venerola vulgaris, has given birth to the opinion, that most cases of excoriation arising from sexual intercourse, are but spurious kinds of the former disease; and this opinion is much strengthened, by their being occasionally followed by the same consecutive diseases.

"The most extensive excoriations I have met with, have appeared to arise from connexion, while the glans and prepuce were in a state of irritation from neglect of cleanliness."

*Diagnosis.* The excoriations arising from different causes are so nearly similar in their appearance and treatment, that diagnosis is of no consequence. Even if confounded with venerola vulgaris, or ulcus induratum, in its earliest stage, the mistake will only last a few days, and is of no practical importance.

*Treatment.* Attention to the bowels; cleanliness of the parts; lotions of the liquor plumbi acet. dilut. in the early stage of the disease. When more advanced, and there is appearance of indolent action in the parts, the application of sulphas cupri or the argentum nitratum, will quicken the cure.

### *Chap. 2. Erysipelas.*

This bears so strong a resemblance to excoriation, that it is almost impossible to distinguish them, excepting by its changing its place from one side of the glans and corona to the other. A wash composed of one part of alcohol to three or four of water is useful; also the black wash, calomel and lime water.*

### *Chap. 3. Venerola Vulgaris, aut Ulcus Elevatum.*

This disease is more frequent than all the other ulcerations put together. It may be seated on any part of the body to

---

* Mr. Evans directs ʒß of calomel to ℥iß of lime water. This is a larger proportion of calomel, we believe, than is generally used in private practice. Rev.

which the cause is applied; but, of course, the body of the penis, the inner or outer surface of the prepuce, and the scrotum are parts most frequently affected. Its progress may be divided into four stages: the pustular, ulceration, elevated, and stage of depression, or cicatrization.

The *first* stage usually commences from three to seven days after connexion, and lasts from four to six days. "It begins with itching and redness, followed by a spherical pustule, surrounded with an areola." The *second* stage is ushered in by the formation of a scab, under which (the ulcerative process still going on) matter sometimes accumulates, and occasions considerable pain. The scab successively enlarging by the exit and concretion of matter, takes a triangular or circular form, varying in colour from yellow to black. If the scab be removed in this stage, a concave ulcer is seen, with a glossy brown, reddish, or dirty yellow colour. The *third* stage (about the eighth day) is marked by an elevation of the edge, and a filling or rising up of the surface of the sore, which eventually becomes seated upon a fungus, raised above the level of the surrounding parts. This elevation of edge and surface is sometimes considerable; the base and edge being usually of a dark red colour, and, until about the fourteenth day, are most frequently surrounded by an efflorescence or areola, especially if the disease be seated on the outer part of the prepuce, or body of the penis. Between the fourteenth and eighteenth day the sore usually rises to its greatest height. The surface of the sore sometimes extends beyond its base, giving the appearance as though a ligature were tied tightly about it, or, as it were, the form of a mushroom.

"When the sore has attained its greatest height, it remains stationary for an uncertain time, after which it gradually, though perhaps very slowly, declines and heals; which process forms the *fourth* stage."

If this disease be situated where there is a certain density of cuticle, there is eventually left a permanent depression, resembling that succeeding variola or vaccina. The appearance of these ulcers is somewhat modified by their locality; yet,

"Wherever may be the seat of these ulcers on the inner part of the prepuce, their characters are seldom doubtful after the ninth day; when, by drawing the skin well back, and making allowance for the form of the parts, the raised edge and surface cannot escape discovery, for though these may not be plainly discernible all round, they will be so on some one side."

The diseased action often extends far beyond the seat of the sore; thus, if leeches be applied to the groin for the reduction of bubo, the wounds made by them will frequently become so many venerolic sores, which will go through their several stages with more or less regularity.

The time required for healing these ulcerations is generally from four to six weeks. Although the disease generally runs its course mildly, yet sometimes a high degree of constitutional irritation takes place, most commonly the forerunner of an eruptive affection.

"The more regularly the local disease goes through its several stages, the less liable is the patient to secondary symptoms."

The *diagnosis* will be best drawn from a comparison of the *symptoms* of the venerola with those of other affections.

"The difference between this disease and chancre consists—first, in not as a general rule being followed by consecutive diseases when mercury is abstained from; secondly, in not requiring the excitement of the mercurial irritation for its cure; and thirdly, in that irritation being either useless or injurious."

*Causes.* This disease almost invariably proceeds from sexual intercourse, and particularly from the application of certain diseased secretions in that act. Mr. Evans relates experiments to prove, *first,* that it may arise from the application of matter from *a sore of the same description;* and *secondly,* states cases to shew that it arises from the application of "*an altered secretion without any breach of surface, or discernible disease in the female organs.*"

"It is the custom of this place (Valenciennes) to have the public women examined at stated periods, and for this purpose a French surgeon is appointed. At these examinations I have frequently been present, and have always been surprised at the small portion of disease to be found among them; at one which I attended, no less than two hundred women of the lowest description, and of course the most frequented by soldiers, were examined, and *not one* case of disease was found among them; nevertheless the military hospitals had, and continued to have, their *usual number* of venereal cases."*

The above shews very clearly how military hospitals may have their usual quota of venereal cases, in spite of all municipal regulations, which, in fact, completely fail in the attainment of their object. Not a doubt, indeed, can remain in the practitioner's mind, respecting the production of genital ulcerations from diseased female secretions; hence the fallacy, as Hunter long ago observed, of the maxim, "quod non habet non dare potest." The circumstance under consideration explains how it is that when one, two, or more men cohabit with the same woman, in a short space of time,

* "By venereal cases ulcerations are alone meant."

the first, and perhaps the second, may escape, while those who follow may contract the disease. The diseased secretions are generated in the course of this unnatural and immoral transaction.

Mr. Evans, in the third place, relates cases to prove that gonorrhœal matter, or the matter capable of producing gonorrhœa, will *also* occasion this disease.

" Two gentlemen had connexion with a girl, the one shortly after the other; one of them contracted venerola vulgaris, the other gonorrhœa; the girl was examined, she had some *discharge* from the parts, but no *ulceration*."

Mr. E. has seen some cases where this disease has appeared to be a *metastasis* of gonorrhœa, and of the ulcus induratum, coming on after the cure of these affections. He relates two cases, which he thinks are " well calculated to excite a suspicion that this disease may arise spontaneously." He doubts, however, the correctness and soundness of such a doctrine of *equivocal generation*.

*Treatment*. It runs a course, in despite of treatment; and except to allay irritation, nothing should be done in the first or second stage. The elevation of the surface of this sore in the third and fourth stages, would seem to indicate the destruction of the elevated portion as a means of quickening the process of cicatrization. Experience has proved that by so doing we shall only put our patients to unnecessary torture. Mercury introduced by friction or otherwise, so as strongly to affect the system, generally retards the cure, and never appears beneficial. The following is an outline of the treatment recommended by our author. In the *first* or pustular stage, which is seldom seen by the surgeon, however, friction is to be guarded against, the bowels to be kept open, and if there be much inflammation, the parts are to be kept cool with liq. plumb. acet. dil.

In the *second* stage, when there is much pain, a warm poultice, continued longer or shorter, according to circumstances. In general, after the removal of the scab by the poultice, the application of ung. ceræ will prevent its re-formation and the recurrence of pain. If the scab be small, and there is no pain, it is best not to remove it.

When the sore happens to be on the *inner* surface of the prepuce, there is generally much more irritation than when situated on a non-secreting surface. In this case a piece of lint kept constantly wet with the liq. plumb. dilut. should be applied to the part. Rest and abstemiousness, to prevent inflammation, swelling, and phymosis, are necessary; paying great attention to the state of the bowels.

In the *fourth* stage, the surface of the sore is to be daily touched with the sulphate of copper, in so light a manner, as to act as a stimulant, not as an escharotic; or the diluted ung. hyd. nit. The weak ointment of verdigris, mercurial ointment, or the common blue pill spread upon lint may be used.

Throughout the whole of the first and second stages, it is in the highest degree beneficial to confine the patient to bed, by which the formation of a bubo will often be prevented, local irritation avoided, and the chance of constitutional affections greatly lessened. If symptomatic fever run high, antiphlogistic measures must be put in force according to the exigency of the case.

### *Chap.* 4. *Venerola Superficialis.*

This is considered by our author to be a variety, or spurious form of the preceding disease. It begins with a vesicle or pustule, which breaking, forms a crust, under which the cuticle is further removed, in a circular or oval form. The surface of the sore, when exposed, is of a healthy red colour, like a blistered part, and level with the surrounding integuments. The size is usually about that of a shilling; but if neglected, ill treated, or the constitutional symptoms run unusually high, it may increase to a much greater extent. In this disease there is always considerable constitutional derangement, and inflammation and ulceration of the tonsils and pharynx, while pains resembling acute rheumatism, accompany or follow it much more frequently than in venerola vulgaris; in short, our author never met with an instance of this disease which was *not* followed by consecutive affections.

*Cause.* Our author has reason to believe that it has no other cause than impure connection. He has seen several instances where it arose from the application of gonorrhœal matter.

*Treatment.* The constitutional symptoms, in this disease, being often of a highly inflammatory type, the most decisive measures should be had recourse to; "for, in this as in the preceding disease, we not only, by these means, shorten the duration of the sore, but, in all probability, lessen the severity of the consecutive disease." The lancet, purgatives, and diaphoretics generally, with topical applications of sedatives or stimulants, according to irritability or sluggishness of the sore, are the means to be employed.

### *Chap.* 5. *Venerola Indurata, aut Ulcus Induratum.*

This Mr. Evans believes to be another variety of venerola vulgaris. In the early stage, this ulcer is so indistinctly marked that it may readily be confounded with several other affections; but sooner or later it assumes its characteristic feature, "the base becoming of a *cartilaginous hardness*, unless the sore should be seated on the glans." When this disease is situated at the duplication of the prepuce behind the glans, or spreads to it, there is always a disposition for the ulceration to extend between the skin and body of the penis, in which case alone it puts on an excavated appearance, it being generally a superficial sore with a plain surface.

"A high degree of constitutional derangement sometimes takes place during the existence of these sores; in such cases mortification to a greater or less extent is no uncommon occurrence. Persons whose constitutions are injured by breathing an unhealthful atmosphere, by spare and unwholesome diet, by a long residence in hot climates, or by dissipation, appear to be most liable to this change in the disease.

"I have known gangrene take place as early as twenty-four hours after the appearance of the disease, and in less than seventy-two hours after connexion."

The hardened spots which these sores occasionally leave behind them are very apt to become again ulcerated, from want of attention to cleanliness, or from irritation by coition, &c.

*Cause.* Though in almost every instance the disease could be traced to impure intercourse, yet our author relates two cases, shewing that the application of gonorrheal or other morbid matter, generated by the person affected, may produce the disease.

*Treatment.* "Extensive inflammation, swelling, and phymosis, are not frequently attendant on this sore, except in cases where mortification takes place, or is about to do so, and even in these, the two last are not always present. When the first occurs, whether accompanied or not by the other two, we must not only make use of the local remedies to reduce it, but as gangrene under these circumstances is in this disease much to be feared, have immediate recourse to more decisive measures.

"In some cases, where there is a disposition to gangrene, a high degree of constitutional irritation takes place; there is great anxiety, heat of skin, increased quickness and fullness of the pulse, a white or furred tongue, great pain of the part, and swelling of the prepuce; when these are present, there is no time for hesitation; every mean must be employed to lower this destructive action; in the accomplishment of which, the lancet will prove, in almost every instance, the only thing upon which we can with safety depend. The other auxiliaries, such as cathartics, diaphoretics, cold applications, and the antiphlogistic regimen, must not, however, be neglected, and the patient, during the time should keep his bed, and his room be well ventilated and kept cool." P. 110.

Sometimes, but not generally, the above strong measures will leave a degree of debility behind requiring bark and opium, especially the latter, for establishing a healthy state both of the constitution and the part. The separation of the sloughs will be accelerated by the application of warm poultices instead of the cold wash; and, not unfrequently, stimulating applications are useful auxiliaries. A change in the colour of the sore, to that of lividity, while the discharge turns the lint black, and has a peculiar fœtid smell, may warn us of approaching gangrene.

"In these cases, the same decisive measures must be adopted for its prevention, as for arresting its progress when commenced." P. 111.

In a few cases, a most tormenting itching in the surrounding parts, where the sloughing action was present, or about to take place, and even after it had been arrested, deprived the patients of rest. An alcohol wash with some laudanum was found very useful, and also the calomel and lime water.

"In cases of mortification, the residence of the patient is a subject of the utmost importance; and, if low and swampy, or badly ventilated, should be changed to a more healthy one.

"Though in general the lancet is the remedy most to be depended on, yet it must not be indiscriminately used, for whenever the pulse is below the standard of health, opposite measures must be employed."

"In ordinary cases, the topical applications made use of in my hospital have been of the simplest nature, such as the weak solution of acetate of lead, or the alcohol wash.

"Extirpation of the hardened part, as recommended by Celsus, has been practised by my friend staff surgeon Murray with the best success." P. 112.

Our analysis has extended so far, that we are unable to notice the subject discussed in the Appendix. We have, however, in this article, given such an analytical view of Mr. Evans's work, as will render its merits appreciable, and its contents fully known in every quarter of the globe to which this Journal travels. Mr. Evans is evidently an accurate observer, a faithful describer, an unprejudiced narrator, and a judicious practitioner. With these qualities and qualifications, we have no doubt but Mr. Evans will complete the work, which he has here commenced, in a manner that will reflect great credit on himself, and confer a lasting benefit on the profession. We shall, therefore, look with some impatience for the second part of our author's researches and observations; nor will we be slow in making them known throughout the medical world.

